# Artificial intelligence in breast cancer diagnostics

**DOI:** 10.1016/j.xcrm.2022.100851

**Published:** 2022-12-20

**Authors:** Caterina AM. La Porta, Stefano Zapperi

**Affiliations:** 1 Department of Environmental Science and Policy, Center for Complexity & Biosystems, University of Milan, via Celoria 10, 20133 Milan, Italy; 2 CNR - Consiglio Nazionale delle Ricerche, Istituto di Biofisica, via Celoria 10, 20133 Milan, Italy; 3 Department of Physics, Center for Complexity & Biosystems, University of Milan, via Celoria 16, 20133 Milan, Italy; 4 CNR - Consiglio Nazionale delle Ricerche, Istituto di Chimica della Materia Condensata e di Tecnologie per l'Energia, Via R. Cozzi 53, 20125 Milano, Italy

## Abstract

Since breast cancer deaths are mainly due to metastasis, predicting the risk that a primary tumor will develop metastasis after a first diagnosis is a central issue that could be addressed by artificial intelligence. To overcome the problem posed by limited availability of standardized datasets, algorithms should include biological insight.

## Main text

### The risk of metastasis in breast cancer

Cancer is one of the leading causes of death in the western world and it is increasing in the developing world. Within cancers, breast cancer takes a terrible toll on women, with 500,000 deaths reported each year. Nowadays, people do not die from the primary tumor but instead die from secondary tumors called metastasis, which account for 90% of tumor mortality. According to World Health Organization (WHO) statistics, about one-third of these cancer fatalities could have been avoided through earlier detection and treatment. A critical barrier to develop effective drugs to treat cancer metastasis is the high heterogeneity of tumor cells implying that each cell of a specific tumor is slightly different from the others.^1^ Plasticity is one of the emerging properties of tumor cells that helps them to escape from a drug’s effects, leading to the development of drug resistance. We discussed this point in a recent book focused on phenotypic switching where we highlighted three important issues^2^: (1) the impact of the environment on the plasticity of the tumor cells; (2) the correlation between senescence and plasticity; and (3) the role of phenotypic switching in inducing collective cell migration. Environment-modulated cancer cell plasticity was clearly shown in different types of tumors, including breast cancer.^3^ This implies the possibility to have dormant cancer cells if the surrounding environment permits it. On the other hand, treatment with specific drugs could help selecting cell subpopulations that will remain dormant, leading to drug resistance. An additional mechanism is provided by senescent cells that can contribute to keep the cells viable for an extended period exhibiting a senescence-associated secretory phenotype, characterized by the release of many factors including proinflammatory cytokines and chemokines, highlighting again the complex interaction between cells and environment. Furthermore, senescent cells can revert their phenotype, resuming their growth.^4^ Finally, since cell migration is a key aspect of aggressiveness, plasticity of cancer invasion and metastasis depends on the ability of cancer cells to switch between collective and single-cell dissemination through the regulation of cadherin-mediated cell-cell junctions.^5^

The strong heterogeneity and plasticity of breast cancer represents a serious issue for an effective treatment, since most currently available drugs are designed to target general biological aspects of the tumors, without considering the specificity of each tumor in each patient. Predicting the individual risk of aggressiveness of primary breast cancer would allow physicians to choose the best therapeutic strategy, limiting overtreatment and side effects that are detrimental for the patient’s quality of life. There is therefore a pressing need to develop predictive tools for personalized therapies that could be more sustainable and economically affordable. This aspect appears particularly urgent in the context of immunotherapy that is very effective in some cases, but also extremely expensive. Clearly it is very important to identify in advance the patients that are most likely to respond. In this respect, artificial intelligence (AI) holds great promise to reach the goal of stratifying breast cancer patients according to the aggressiveness of their specific tumor, their individual risk of metastasis, and their likelihood to respond to a given therapy.

### Can AI predict the future?

We can identify two main pathways for the application of AI to breast cancer diagnostics, the first relying on image analysis and the second on molecular data. State-of-the-art deep learning algorithms, when trained with large datasets of annotated images, enable very precise image classification and can easily be deployed on histological images. In the context of breast cancer, deep learning methods are able to reliably assess whether a histological image is referring to a normal tissue, a benign tumor, *in situ* carcinoma, or an invasive carcinoma.^6^ While these tools show great promise in assisting the pathologist in the diagnosis after biopsy, we are still far from an accurate classification of the metastatic risk or to predict the likelihood to respond to a specific treatment. It is not clear if it will ever be possible to extract such a fine-grained information from images alone, even if we increase the training set by collecting more images. Tissue morphology might not display enough features to enable a precise prediction of clinical outcome and images might suffer from technical biases due to preparation protocols that might vary among institutions. Furthermore, in order to apply these methods, it will be necessary to build up large and accessible databases of digital images for training the algorithm. Efforts along these lines are underway in many western countries but we are still far from reaching this goal globally.

While there is a large literature investigating genomic mutations, we focus here on gene expression data, which represent (in our opinion) a more promising area of study. Since tumor cells are plastic, gene expression data provide a detailed fingerprint of the tumor at a particular moment in time. One can thus conceive that the expression level of all the genes could encode important information on the phenotype of cancer cells which could be exploited to make predictions. Molecular subtyping of breast cancer is already well established and is based on the expression of of estrogen receptor (ER), progesteron receptor (PR), the human epidermal growth factor receptor 2 (HER2), and the proliferation marker Ki67. Combination of these factors leads to four standard subtypes: Luminal A (ER^+^ and/or PR^+^, HER2^−^, Ki67^low^), Luminal B (ER^+^ and/or PR^+^, HER2^−^, Ki67^high^), HER2 positive (HER2^+^), and triple negative (ER^−^, PR^−^, HER2^−^). While standard clinical guidelines are associated with each of these subtypes, a large heterogeneity is present within each subtype. Due to the growing availability of transcriptomic data for breast cancer, AI methods have increasingly been used to better stratify patients within each molecular subtype.

Early studies applied machine learning methods to the whole transcriptome with the aim of identifying patients with higher risk of tumor relapse and low rate of survival.^7^^,^^8^ The studies focused on the Luminal A subtype and identified a list of genes that, according to the algorithms, best correlated with clinical outcome in the training set. The list was then used to establish a classifier that could be used to screen new patients after validation in a test set. In a similar spirit, a widespread approach to stratify triple-negative breast cancer is based on K-means clustering of whole transcriptomic data, resulting in the establishment of 6 subgroups showing differential response to treatment but limited differences in terms of relapse-free survival.^9^

Unfortunately, these kinds of brute force algorithms suffer from serious problems mainly due to the relative shortage of data available for training. A transcriptome contains roughly N_g_ = 20,000 genes and each of them represents potentially a relevant feature to predict clinical outcome. The number of samples (N_s_) used to train the machine learning algorithm is, however, typically much smaller than N_g_, ranging most of the times to a few hundred for each breast cancer subtype. This problem is well known as the “curse of dimensionality.” When the dimensionality of the objects under study increases, the available data become effectively sparse. Reliable results can often be obtained only with a training set that is much larger than the dimensionality of the object in order to ensure that there are several samples for each combination of gene values. In practice, we would need to train a classifier using tens of thousands of gene expression data to obtain a reliable classification.^10^

A concrete and vivid example of the problems caused by the high dimensionality of transcriptomic data is provided by earlier classification attempts of Luminal A patients based on machine learning,^7^^,^^8^ which we mentioned above. As pointed out by Drier et al.,^11^ the gene lists obtained by two independent studies using two independent patient groups but similar machine learning algorithms showed no overlap. This observation calls into question the reliability of the methodology used to establish the gene lists in the first place. It turns out that the limited success that these methods still achieve in stratifying the patient’s clinical outcome results from basic differences in the proliferation capabilities of the tumors. Proliferation correlates with the activity of many genes in the transcriptome and therefore the activity of virtually any set of genes can be used to stratify patients. In the case of Luminal A, we do not need AI to stratify patients—indeed, proliferation-related marker genes are commonly used to this end. Unfortunately, a similar strategy is not applicable to the other breast cancer subtypes where aggressiveness depends on more than cell proliferation.

We should also mention that the issue of patient stratification is further complicated by the presence of “batch effects,” which prevent the straightforward merging of datasets obtained in different experiments. Experimental details, such as the protocol followed to collect the samples and to extract the genetic material or the platform used to sequence it, can have an important effect on gene expression data, hiding the true biological variability of the dataset. If batch effects are not removed by suitable algorithms,^12^ an AI algorithm may classify the samples according to their batch rather than their biological characteristics, providing results that would be of little practical use.

Since adding different datasets coming from different studies is problematic and the number of available samples N_s_ is often limited, an alternative strategy is to reduce the effective dimension N_g_ of the transcriptome by shifting the attention from genes to pathways. A pathway is a relatively small set of genes working together for a given biological function. Since the number of genes in a pathway rarely exceeds N_g_ = 100, with a number of samples N_s_ > 100 one can overcome the curse of dimensionality. The idea was pioneered by Eytan Domany and his group who introduced pathway deregulation scores (PDS) as a method to identify which pathways are deregulated in individual breast cancer patients.^13^ The method quantifies the overall deregulation of each pathway with respect to a reference sample by fitting a non-parametric, non-linear one-dimensional principal curve through the subspace of the transcriptome defined by the genes of the pathway. PDS can be computed for all known pathways, providing a more coarse-grained picture of the transcriptome of an individual. Clustering algorithms where applied to the PDS scores of breast cancer patients reveal new patient classes with specific drug response and survival statistics.^12^

### Guiding artificial intelligence with biological insight

From our discussion, it should now be clear that while AI methods based on artificial neural networks are incredibly powerful in many domains, including medicine, their straightforward application to disentangle cancer heterogeneity faces important challenges. To fully exploit the potential of AI in providing reliable breast cancer patient stratification strategies that can predict the individual response to a specific treatment or the risk of metastasis and survival, we would need extremely numerous and homogeneous data for training. Such data are at present unfortunately not available. It is, however, still possible to make important progress with the data we have, but we should move away from brute-force black-box type algorithms and exploit the large trove of biological knowledge accumulated in the past decades to design smarter and more targeted algorithms for patient stratification.

We followed this strategy in recent years by developing ARIADNE, an algorithm to stratify the aggressiveness of the tumor in triple-negative breast cancer patients, based on their gene expression data.^14^ The biological observation underlying the algorithm is related to the epithelial-mesenchymal transition (EMT), which describes how polarized epithelial (E) cells transform into mesenchymal (M) cells by down-regulating intracellular adhesion molecules and promoting cell polarity. EMT can sometimes give rise to hybrid E/M cells that display features of both E and M phenotypes, leading to collective invasive capability and increased aggressiveness of the tumor. The EMT is regulated by a complex network involving several genes (N_g_ = 72), that we have recapitulated *in silico* by a Boolean network model.^15^ The model provides a landscape of all possible cell phenotypes that can be used to as a reference map for gene expression data coming from individual breast cancer patients. ARIADNE can perform the projection from gene expression data to the landscape and allows us to identify the patients whose tumor contain a signature of aggressive hybrid states. Notice that these hybrid states could not be identified by measuring the expression of a set of genes but only by considering interactions among genes within the gene regulatory network.^15^ Cross validation with clinical data (with N_s_ > 500) confirmed that the high-risk triple-negative breast cancer patients identified by the ARIADNE algorithm indeed show a higher risk or relapse and low survival.^14^ While the algorithm has been validated with triple-negative breast cancer patients, the strategy is fully general and could readily be extended to other breast cancer subtypes and potentially also other tumors.

### Conclusions

Our discussion of recent applications of AI to breast cancer diagnostics suggests that the most effective strategies use a combination of algorithmic ingenuity and biological insight. Out-of-the-box AI algorithms are widely available and are extremely successful in many different areas where large-scale datasets for training are available. Straightforward application of these algorithms to stratify breast cancer patients is hampered by the limited number of available transcriptomic data. On the other hand, the deployment of deep learning algorithms to histological images has provided a promising diagnostic tool for early breast cancer detection that is likely to improve further thanks to the growing availability of images needed for training. It is unclear, however, if histological images include enough information to discriminate the future evolution of the tumor. Combining images with gene expression data might lead to interesting developments in the near future.
